# Tissue-Level Cardiac Electrophysiology Studied in Murine Myocardium Using a Microelectrode Array: Autonomic and Thermal Modulation

**DOI:** 10.1007/s00232-017-9973-y

**Published:** 2017-08-01

**Authors:** Jem D. Lane, David Montaigne, Andrew Tinker

**Affiliations:** 1 William Harvey Heart Centre, Barts & The London School of Medicine and Dentistry, London, UK; 2 Department of Cardiac Functional Investigations, CHU Lille, 59000 Lille, France; 3 Univ. Lille, 59000 Lille, France; 4 Inserm, UMR 1011, 59000 Lille, France; 5 Institut Pasteur de Lille, Lille, France; 6 European Genomic Institute for Diabetes (E.G.I.D.), FR 3508, 59000 Lille, France

**Keywords:** Autonomic regulation, Cardiac electrophysiology, Temperature

## Abstract

Cardiac electrophysiology is regulated by the autonomic nervous system, and this has both pathophysiological, and possibly therapeutic importance. Furthermore, chamber differences in electrophysiology exist between atria and ventricles, yet there have been few direct comparisons. There is substantial literature on ion channel modulation at the single-cell level but less work on how this affects tissue-level parameters. We used a microelectrode array system to explore these issues using murine atrial and ventricular tissue slices. Activation time, conduction velocity and repolarisation were measured, and their modulation by temperature and pharmacological autonomic agonists were assessed. The system recorded reliable measurements under control conditions in the absence of drug/thermal challenge, and significant baseline differences were found in chamber electrophysiology. The sodium channel blocker mexiletine, produced large magnitude changes in all three measured parameters. Carbachol and isoprenaline induced differing effects in atria and ventricles, whereas temperature produced similar effects on activation and repolarisation.

## Introduction

Great progress has been made in our understanding of cardiac electrophysiology over the last century (Janse and Rosen 2006). The advent of patch clamping allowed the study of single cardiomyocytes and led to the isolation of specific currents underlying the cardiac action potential (Coraboeuf and Weidmann 1949; Ling and Gerard 1949). This, combined with the cloning of ion channel subunits, led to an explosion of knowledge about channel regulation often in exquisite molecular detail. However, there has been comparatively less study of the electrophysiological properties of intact tissues and the hormonal and thermal regulation of these. The key difference compared to single-cell systems is that intercellular connections are preserved that are critical in regulating the three key electrophysiological parameters—activation, repolarisation and conduction. Modulation of these occurs physiologically via the autonomic nervous system. Sympathovagal imbalance can perturb them, however, creating conditions favourable to arrhythmogenesis (Shen and Zipes 2014). And while little attention has been paid to thermal modulation, its importance is illustrated by the role of fever in triggering arrhythmias in Brugada syndrome, for example.

Microelectrode arrays (MEA) have been used to study cardiac tissue slices for just over a decade (Pillekamp et al. 2005). This system offers the advantages of being able to assess drug effects in tissue, as opposed to single cells. Multiple slices from the same heart can be assessed, maximising use of the animal and possibly reducing variability between slices. Few studies have directly compared electrophysiological properties in atria and ventricles (Giles and Imaizumi 1988). Important differences may exist which could be amenable to exploitation with drugs and if adapted robustly to the mouse may be suitable for examining genetically modified strains. We used the MEA to evaluate the effects of autonomic modulators and temperature on ex vivo murine cardiac atrial and ventricular tissue slices, and to compare the responses in preparations from each chamber.

## Methods

### Experimental Animals

Mice were housed in individually ventilated cages, with free access to standard rodent chow and water, with 12-h day–night cycles, and in accordance with British Home Office animal welfare guidelines (PPL 70/7665). All mice were on a 129 Sv background; males and females were used. At the time of study, they were 8–12 weeks old, weight over 20 g.

### Buffer Solutions

The following modified Krebs–Henseleit buffer (hereafter termed ‘Krebs’) master solution was made up prior to slice preparation on the day of procedure (molecular concentration): NaCl (118 mM), KCl (3.8 mM), MgSO_4_·7H_2_0 (1.19 mM), NaHCO_3_ (25 mM), KH_2_PO_4_ (1.19 mM), d-glucose (5 mM) and sodium pyruvate (2 mM) were dissolved in 1 L of distilled water (all from Sigma-Aldrich). This ‘Calcium-free Krebs’ solution was used to produce the following modified Krebs solution, with addition of appropriate amounts of CaCl_2_, KCl and 2,3-butanedione monoxime (BDM) (Sigma-Aldrich): Normal Krebs (Ca^2+^ 1.4 mM, K^+^ 5 mM) at 4 °C, Perfusion-Slicing Krebs (Ca^2+^ 0.6 mM, K^+^ 15 mM, BDM (10 mM) at 4 °C, Resting Krebs (same as Perfusion-Slicing Krebs) at 21 °C, Preparation Krebs (Ca^2+^ 1.4 mM, K^+^ 5 mM, BDM 10 mM) at 21 °C and Bathing Krebs (Ca^2+^ 1.4 mM, K^+^ 5 mM) at 37 °C.

### Measurement of Myocardial Electrophysiology Using Microelectrode Arrays

Using a microelectrode array (MEA, Multichannel Systems), we investigated, in ex vivo murine left atrial and ventricular tissue preparations, the effects of temperature and pharmacological agonists on electrophysiological (EP) parameters, i.e., activation time (AT) latency, effective refractory period (ERP) and conduction velocity (CV). Isolated mouse hearts were washed in Normal Krebs, before being mounted on a Langendorff setup and perfused with Perfusion-Slicing Krebs. The heart was transected at the atrioventricular groove, and atrial tissue was transferred directly to the MEA without tissue slicing and perfused with Bathing Krebs solution (37 °C; 95% oxygen/5% carbon dioxide).

The left ventricular slices were prepared for slicing in a similar manner to that described previously (Halbach et al. 2006). Briefly, the ventricular base was glued to a cylindrical metal ‘coin’, and placed in a chamber on the vibratome (Vibrating Microtome 7000-smz2, Campden Instruments Ltd, UK). The chamber was filled with Perfusion-Slicing Krebs at 4 °C, After removal of the apex, three slices of thickness 250 μm were cut with slicing settings of frequency 50 Hz, amplitude 1.00 mm, z-deflection <2 μm and advance rate 0.02 mm/s. This resulted in a slicing plane similar to the ‘horizontal transmural’ slices described in Bussek et al’s paper (Bussek et al. 2009). After each slice was cut, it was transferred to a Falcon tube containing carbogenated (95% oxygen, 5% carbon dioxide) Resting Krebs at room temperature. Slices were kept in Resting Krebs for 30 min, after which they were transferred to carbogenated Preparation Krebs also at room temperature.

Left atrial and ventricular electrophysiological parameters were assessed during electrical stimulation using a microelectrode array (MEA) system which allowed non-invasive synchronous multifocal recording of unipolar electrograms (UEGMs), referred to as extracellular field potentials in the literature. The MEA (MEA2100, Multi Channel Systems, Reutlingen, Germany) consists of 60 microelectrodes arranged in an 8 × 8 matrix, with a 20-μm electrode diameter and an interelectrode distance of 200 μm. Myocardial samples were positioned in the centre of the MEA dish, held in contact with electrodes by a holder, and continuously superfused with oxygenated Bathing Krebs solution at 37 °C. For ventricular slices, a run-in period of 30 min was used to allow washout of BDM, and recovery of excitability. During this time, the slice was paced at 1 Hz for 20 min, then 2 Hz for 10–15 min, before checking threshold and commencing stimulation at 4 Hz. For the experimental protocol, electrical stimulation (biphasic pulses, twice current threshold, 2 ms duration, 4 Hz frequency) was applied via one of the microelectrodes. UEGM data were acquired simultaneously from all 60 microelectrodes with MC Rack (Multi Channel Systems, Reutlingen, Germany). S1–S2 train stimulation with a S1–S1 cycle length of 250 ms and S1–S2 starting at 140 ms, was used to assess effective refractory period (ERP).

To assess conduction properties, tissue samples were stimulated at 4 Hz, and the UEGMs obtained were processed using LabChart7 (ADInstruments, UK) to define local activation time (LAT) based on the most negative derivative of the UEGM (details below). Average conduction velocity (CV) was calculated using interelectrode distance and the difference in activation times, as previously described (Opel et al. 2015) (see below).

Electrophysiological parameters were assessed in a series of experiments: (i) under control conditions in the absence of pharmacological challenge over 35 min; (ii) at baseline and in the presence of the muscarinic agonist carbachol (Alfa Aesar) at concentrations between 1 nm and 1 μm, followed by reversal with the receptor antagonist atropine to demonstrate continued responsiveness; (iii) at baseline and in the presence of the non-specific β-adrenoceptor (βAR) agonist isoprenaline (Sigma-Aldrich), at concentrations between 1 nm and 1 μm, followed by reversal with the beta adrenoceptor antagonist propranolol to demonstrate continued responsiveness; (iv) during a temperature challenge, at 37, 34 and 40 °C, achieved by varying the temperature of Krebs perfusate sequentially; (v) at baseline and in the presence of mexiletine (Sigma-Aldrich) at 10 and 100 μm.

### Signal Analysis—Calculation of Local Activation Time and Conduction Velocity

**Fig. 1:**
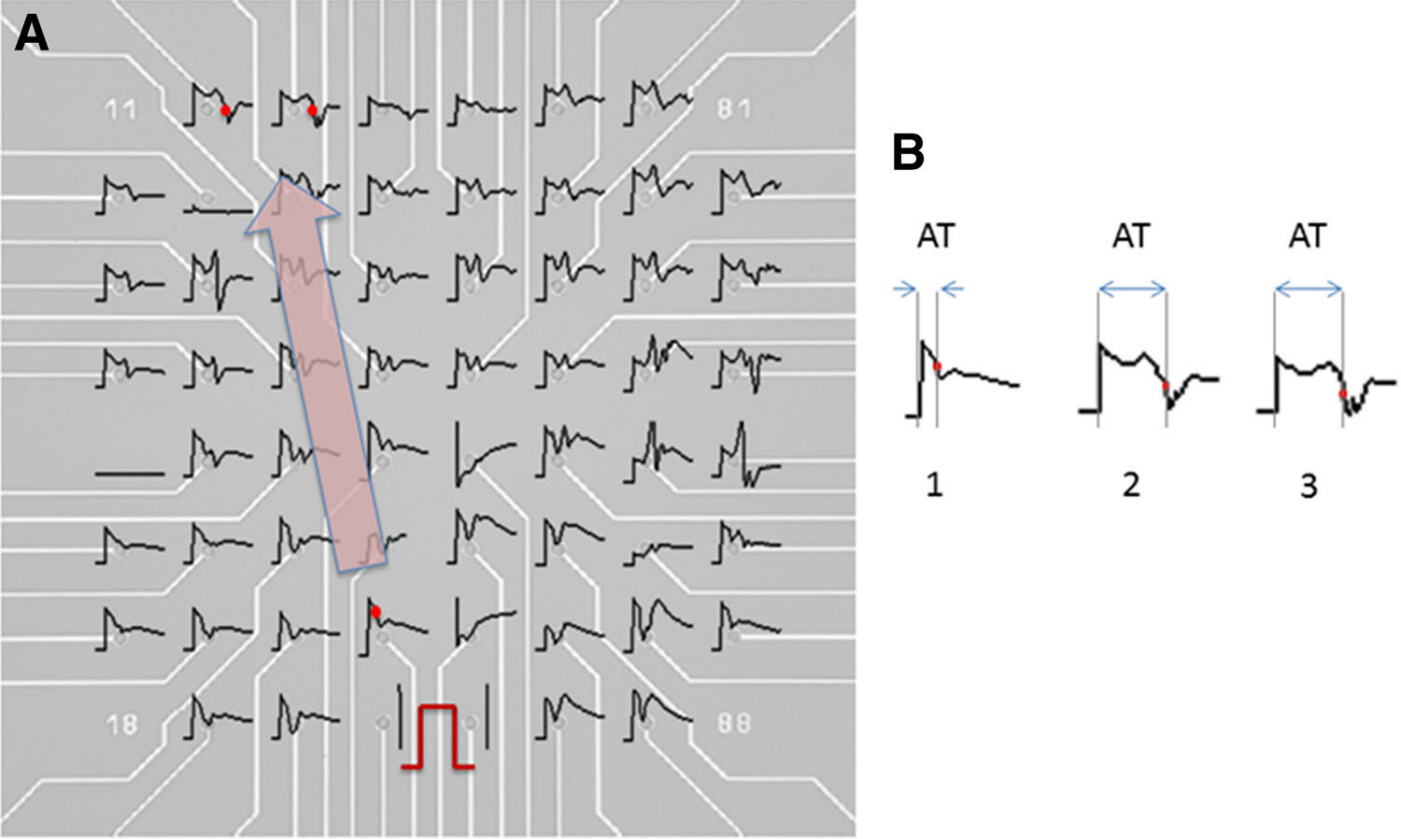
Representative UEGMs recorded from a ventricular tissue slice using the MEA. **a** The square wave (*red*) represents the stimulus at a peripheral electrode. The proximal *red dot* is the negative deflection on the nearest (reference) electrode to the stimulus. Linear conduction is assumed, and the AT at two distal electrodes on the opposite side of the MEA is marked by the *red dots*, with the *arrow* indicating direction of propagation. **b** Magnified UEGMs from **a**. AT is measured from the stimulus onset to the steepest part of the negative deflection, marked by a *red dot*. (*1*) was recorded at the reference electrode, and (*2*, *3*) from the distal electrodes (Color figure online)

**Fig. 2:**
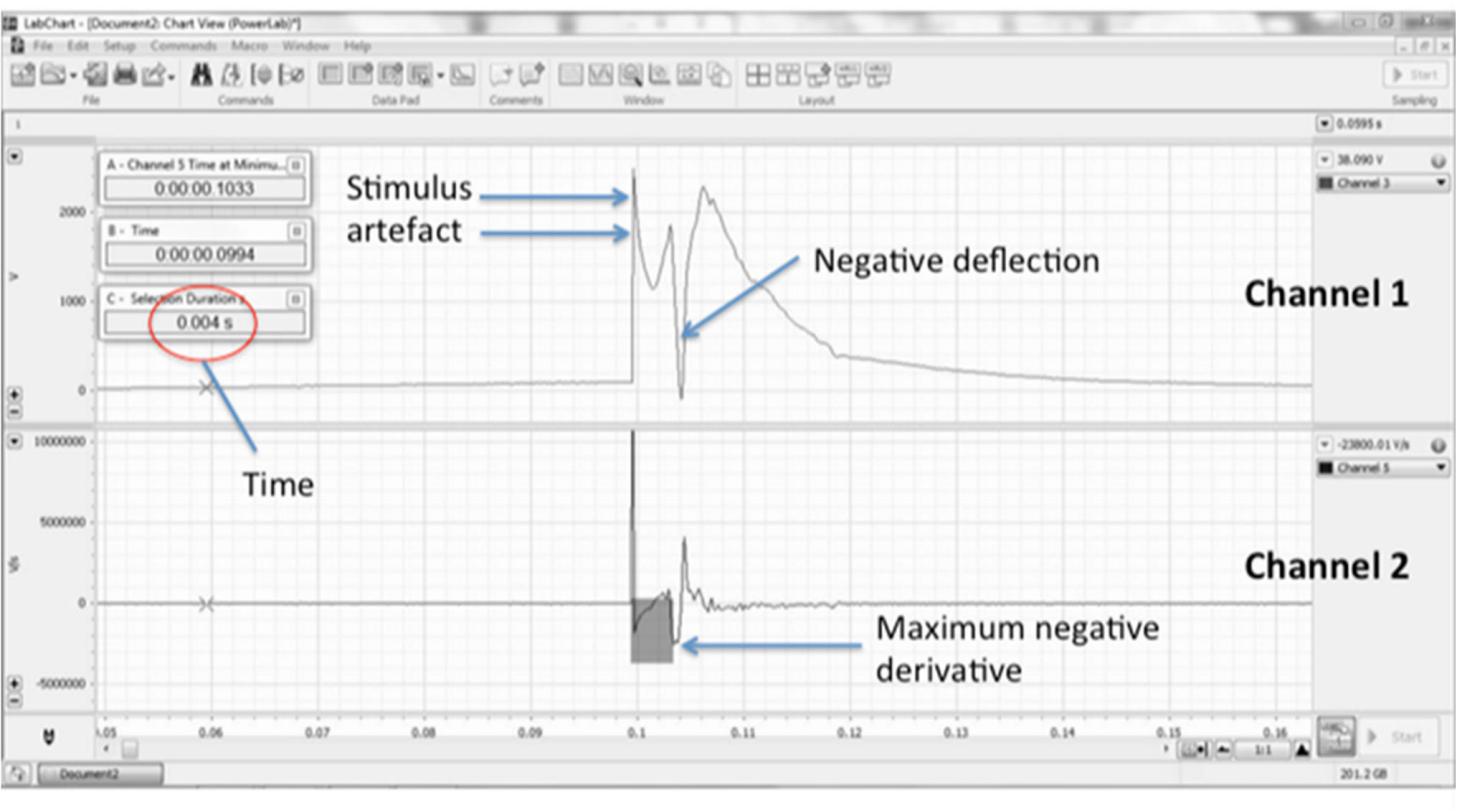
Measurement of activation time. In Channel 1, the UEGM is shown with stimulus artefact and negative deflection. Channel 2 displays the first derivative of Channel 1. AT is measured from the stimulus artefact to the maximum negative derivative (*grey shaded area*). This time is shown in the *red circle* (Color figure online)

The method of analysis described below assumed linear impulse propagation across the MEA from the reference electrode (the one within 400 μm of the stimulating electrode used to define earliest capture; see Fig. 1) to distal electrodes at which activation times were measured. In LabChart, activation time in milliseconds at the reference electrode was measured from the commencement of the stimulus artefact to the maximum negative value on the derivative channel (Fig. 2). The process was repeated for the distal electrode channels to determine their activation times. The distance between the reference electrode and each distal electrode was calculated, and conduction velocity derived from these values. Velocities <10 cm/s or >150 cm/s were excluded from analysis, as they were felt to be outside what could be considered normal.

### Statistical Analysis

All statistical analysis was performed with StataIC 12 (StataCorp LP, USA). Multilevel mixed-effects models were employed to analyse repeated measures data. Statistical significance for was assumed if *p* < 0.05 in all circumstances.

## Results

### Pooled Baseline Data

Table 1 summarises the baseline values for left atrial (LA) and left ventricular (LV) ERP, CV and LAT from all experiments, i.e., the measurements prior to any pharmacological or thermal challenge. The value of each parameter was lower for atrial tissue than ventricular, and these differences were highly significant: *p* = 0.004 for ERP, *p* = 0.003 for CV and *p* < 0.001 for LAT. However, there was variability between individual experiments and in some smaller subsets of experiments, these differences were not apparent (see below).

**Table 1:** Baseline values for atrial and ventricular ERP, CV and LAT

Variable	*n*	Mean	SD	25th centile	Median	75th centile
Atrial ERP (ms)	28	43.04	12.87	31.5	39	53.5
Ventricular ERP (ms)	53	55.96	21.36	38	56	66
Atrial CV (cm s^−1^)	28	37.18	8.84	33.5	38	41
Ventricular CV (cm s^−1^)	36	53.94	27.27	35.5	48	59
Atrial LAT (ms)	28	0.63	0.18	0.5	0.6	0.75
Ventricular LAT (ms)	36	1.94	1.01	1.3	1.8	2.2

*n* Number of samples

### EP Parameters in LA and LV Myocardium in Control Conditions

**Fig. 3:**
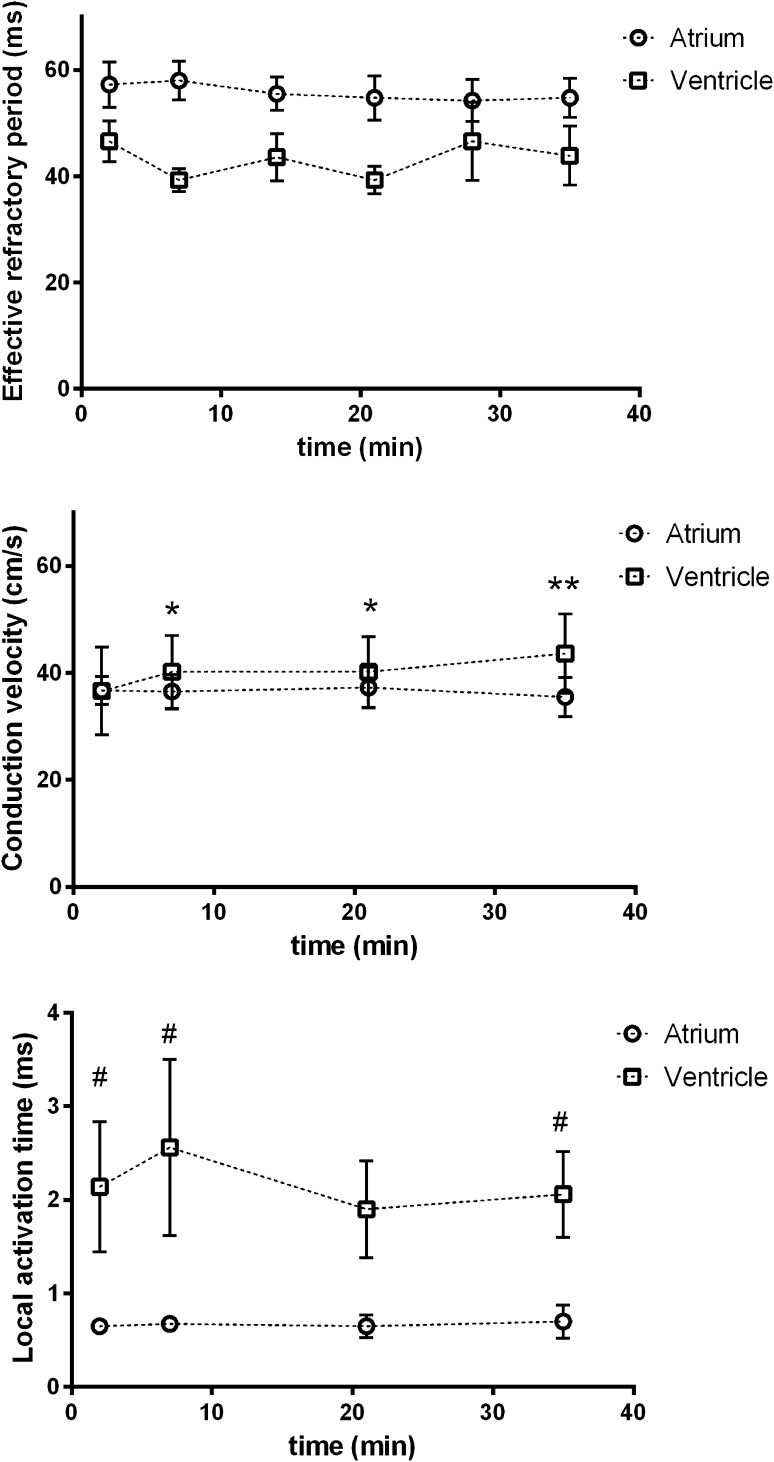
Stability of measurements with time. There were no differences in ERP between groups at any time point during the protocol (*n* = 4 atria; *n* = 8 ventricles). And compared to baseline, there were no differences at any time point for either group. For CV, there was no significant difference between groups at baseline (*p* = 0.985), nor at any time point (*n* = 4 atria; *n* = 5 ventricles). Compared to baseline, atria did not differ at any time. Ventricles were significantly different at 7 min (+3.6 cm/s, *p = 0.028) and 21 min (+3.6 cm/s, *p = 0.028), and highly significantly different at 35 min (+7 cm/s, **p < 0.001). There were significant between-group differences in LAT at baseline (Δ1.4 ms, ^#^ *p* = 0.030), at 7 min (Δ1.89 ms, ^#^ *p* = 0.006) and 35 min (Δ1.36 ms, ^#^ *p* = 0.047) (*n* = 4 atria; *n* = 5 ventricles). Compared to baseline, there were no significant differences for either group at any time

It is important to establish how stable the recordings are with time and this might indicate deterioration of the tissue. Thus, in a subset of experiments, we studied the stability of the measurements with time. As shown in Fig. 3 and taken together, the measured EP parameters (ERP, CV and LAT) were stable in both atrial and ventricular *ex vivo* myocardial preparations for at least 30 min.

### Response of EP Parameters in LA and LV Myocardium to Changes in Temperature

**Fig. 4:**
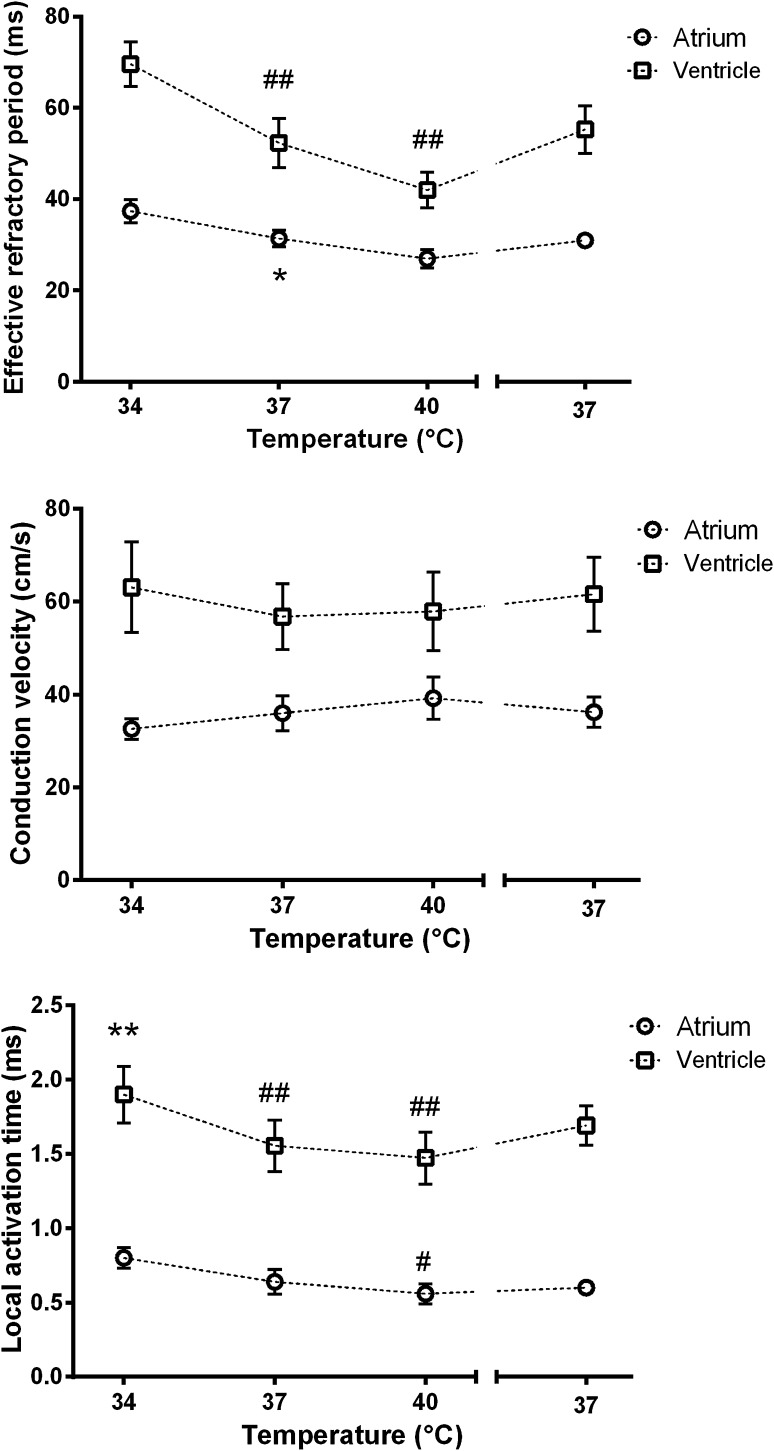
Response of EP parameters to temperature. Mean atrial ERP at 37 °C was 31 ms, and mean ventricular ERP was 52 ms (*p = 0.015). Compared to 34 °C, atrial ERP was not significantly different at 37 °C, though it approached significance at 40 °C (*p* = 0.058). Compared to 34 °C, ventricular ERP was highly significantly different at 37 °C and 40 °C (−17 ms and −28 ms, respectively, ^##^ *p* < 0.001 for both). At 37 °C, mean atrial CV was 36 cm/s, and mean ventricular CV was 57 cm/s (*p* = 0.088). Compared to 34 °C, there were no significant differences at 37 or 40 °C for either atria or ventricles. Mean atrial LAT at 37 °C was 0.6 ms, and mean ventricular LAT was 1.6 ms (**p < 0.001). Compared to 34 °C, atrial LAT did not differ significantly at 37 °C, but did differ at 40 °C (^#^ *p* = 0.022). Ventricular LAT was highly significantly different at 37 °C and at 40 °C (^##^ *p* < 0.001 for both)

We next moved on to assess how the electrophysiology of tissue slices from atria and ventricles responded to temperature and the results of the thermal challenge experiments are shown in Fig. 4. Taken together, temperature had effects of reasonable magnitude on both ERP and LAT in both atrial and ventricular tissues, with shortening of ERP and LAT as temperature increases. CV however was not affected.

### Response of EP Parameters in LA and LV Myocardium to Muscarinic Receptor Agonist

**Fig. 5:**
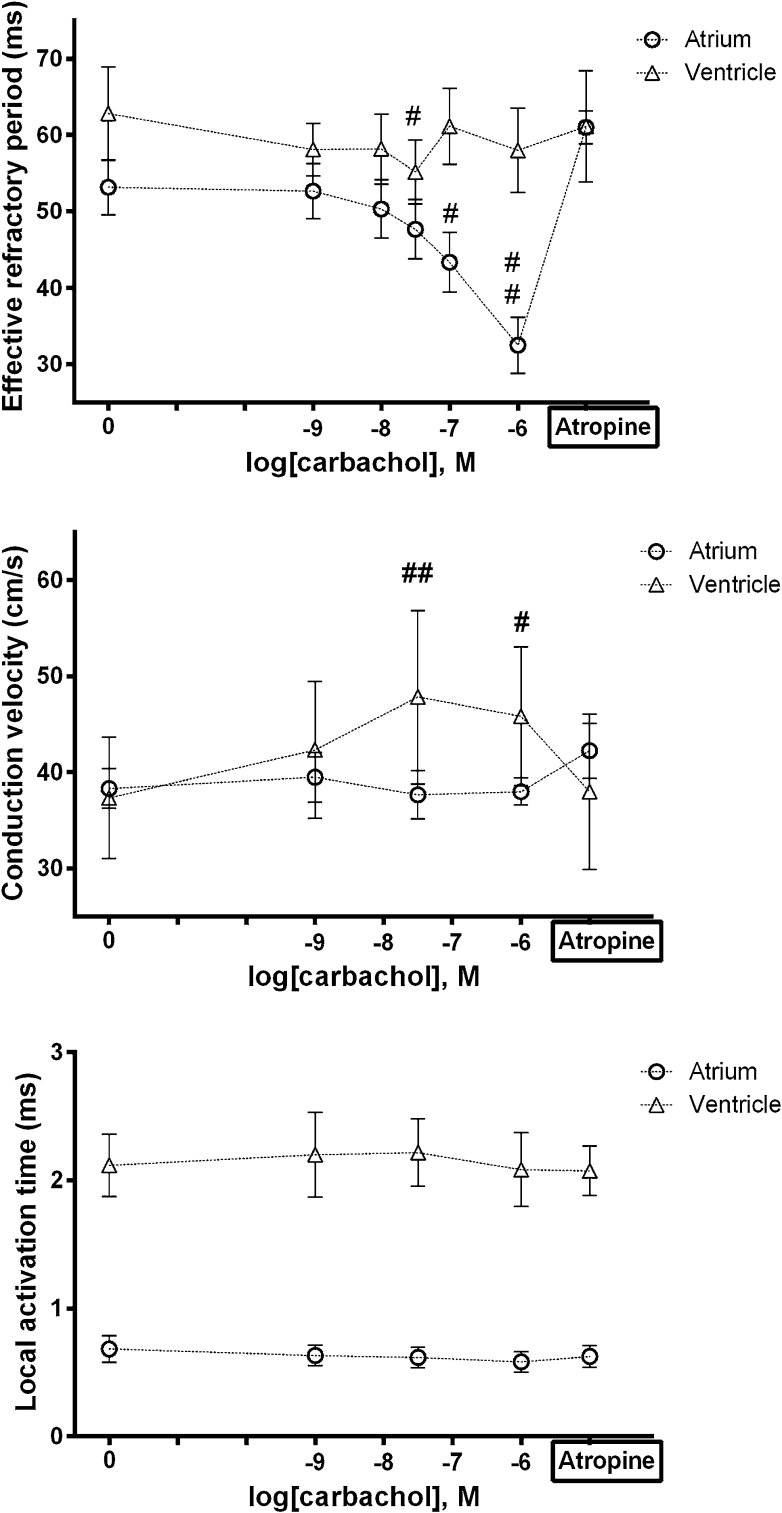
Response of EP parameters to carbachol. Compared to baseline, atria had significantly shorter ERP at 10^−7^ M (−10 ms, ^#^ *p* = 0.042) and 10^−6^ M carbachol (−21 ms, ^##^ *p* < 0.001). For ventricles, ERP was significantly shorter at 3 × 10^−8^ M (−8 ms, ^#^ *p* = 0.032), but did not differ at any other concentrations. Compared to baseline, ventricular CV was highly significantly faster at 3 × 10^−8^ M (+11 cm/s, ^##^ *p* = 0.001) and at 10^−6^ M (+8.5 cm/s, ^#^ *p* = 0.009). There were no differences for atrial CVs. Compared to baseline, there were no significant changes for either chamber in LAT at any concentration of carbachol

We examined the changes of ERP, CV and LAT to challenge with a muscarinic receptor agonist namely carbachol. Overall, both atrial and ventricular tissues responded to muscarinic agonists, yet in a different manner (Fig. 5): carbachol had no effect on ERP in the ventricle, whereas it modestly but significantly increased CV in this chamber. Conversely, in the atrium it had a large effect on ERP but no effect on CV.

### Response of EP Parameters in LA and LV Myocardium to Beta-Adrenergic Receptor Agonist

**Fig. 6:**
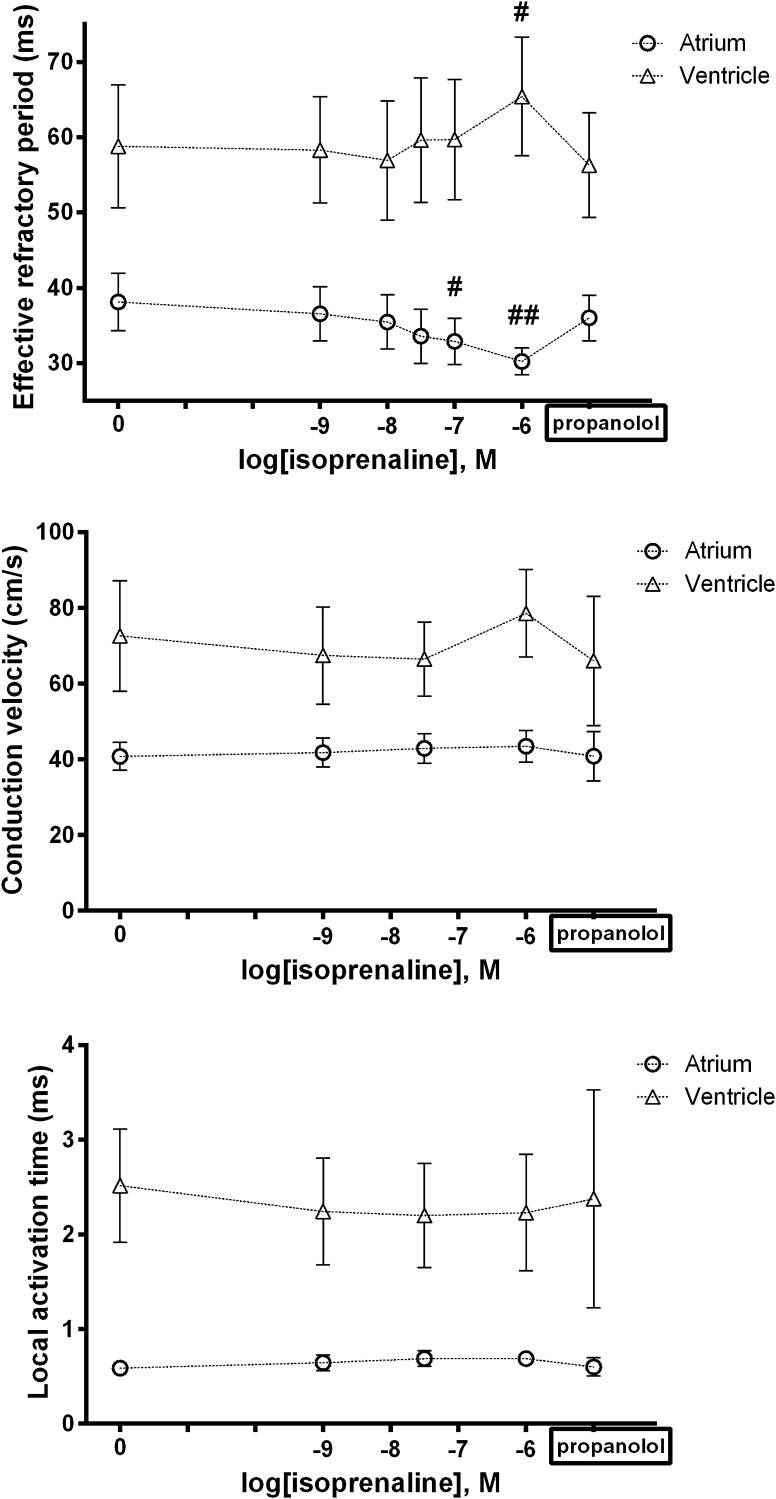
Response of EP parameters to isoprenaline. Compared to baseline, atrial ERP was significantly shorter at 10^−7^ M (−5 ms, ^#^ *p* = 0.028) and 10^−6^ M isoprenaline (−8 ms, ^##^ *p* = 0.001); in contrast, ventricular ERP was highly significantly longer at 10^−6^ M only (+7 ms, ^#^ *p* = 0.004). There were no significant differences in CV with isoprenaline, compared to baseline, for either chamber. And neither were there any differences for atrial LAT at any concentration. For ventricles, however, there were small but significant reductions compared to baseline at each concentration

We examined the changes of ERP, CV and LAT to challenge with a b-adrenergic receptor agonist namely isoprenaline. Taken together, isoprenaline had interesting opposing effects on atrial and ventricular ERP, with a shortening of ERP in atrial and prolongation in ventricular slices (Fig. 6). Isoprenaline did not have any impact on CV in either cardiac chamber but had slight effects on ventricular LAT.

### Response of EP Parameters in LA and LV Myocardium to a Class I Anti-arrhythmic Drug

**Fig. 7:**
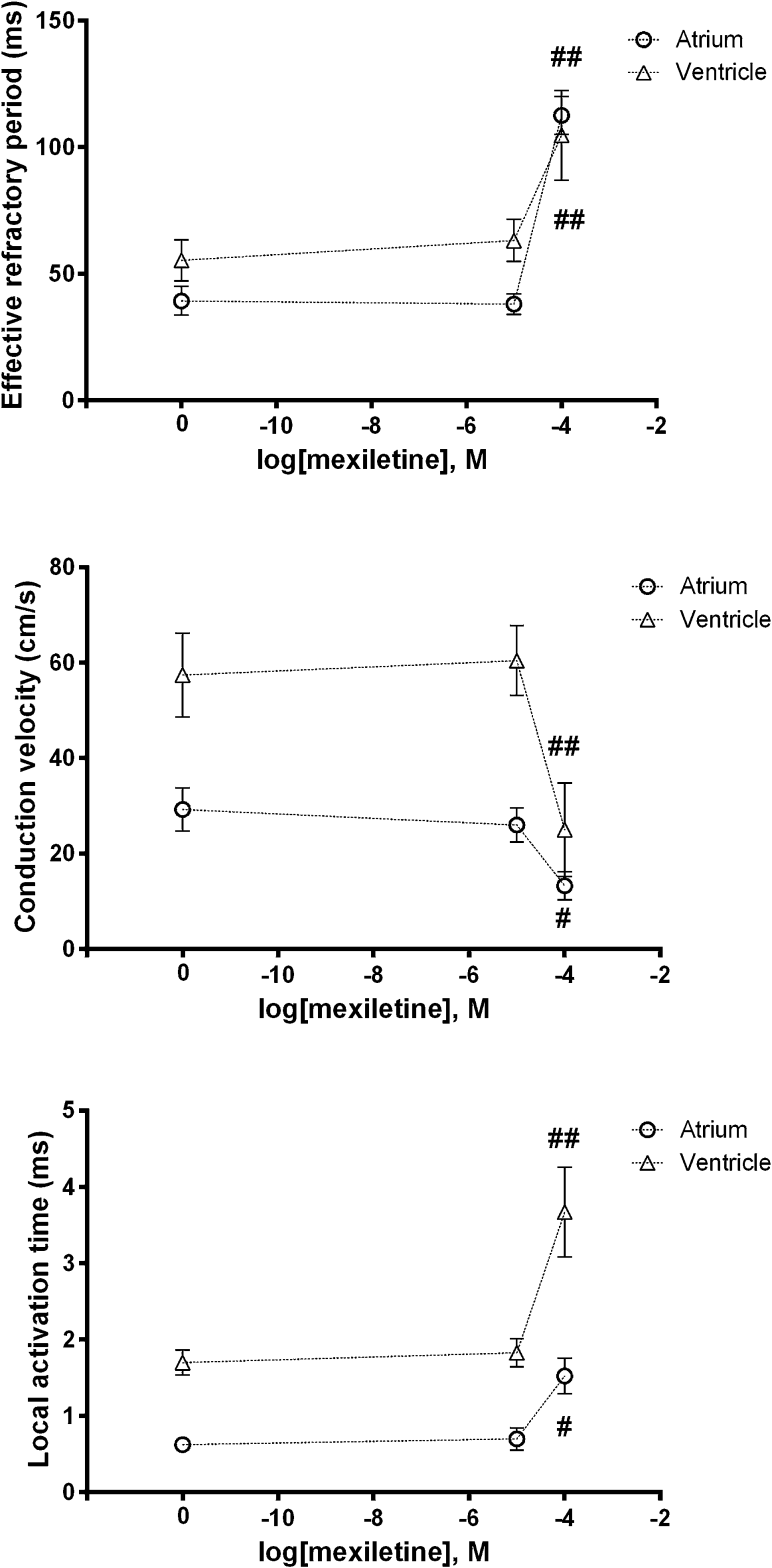
Response of EP parameters to mexiletine. Compared to baseline, there were no differences in ERP at 10^−5^ M mexiletine for either chamber, though there were highly significant differences (^##^ *p* < 0.001) for both atria and ventricles at 10^−4^ M (+73 ms and +49 ms, respectively). And there were no differences in CV for either group at 10^−5^ M compared to baseline, but at 10^−4^ M atrial CV was significantly lower (-16 cm/s, ^#^ *p* = 0.019), and ventricular CV was highly significantly lower (−32 cm/s, ^##^ *p* < 0.001). There was no difference at 10^−5^ M mexiletine compared to baseline, in either atrial or ventricular LAT. But atrial LAT was significantly longer at 10^−4^ M (+0.9 ms, ^#^ *p* = 0.017) and ventricular LAT was highly significantly longer (+2.0 ms, ^##^ *p* < 0.001)

Finally, we used a well-established pharmacological agent that should have predictable effects on tissue electrophysiology (Fig. 7). Overall, mexiletine affected both atrial and ventricular EP parameters in a similar manner, resulting in an expected increase in ERP and LAT, and decrease in CV.

## Discussion

Arrhythmias are a major cause of morbidity and mortality. Currently, drugs, ablation and implantable cardioverter defibrillators are the mainstays of treatment. Although there are similarities in the mechanisms of atrial and ventricular arrhythmias, differences in anatomy and ion channel expression exist, and are potentially exploitable in terms of therapies. The autonomic nervous system has been implicated in initiating arrhythmias, yet our understanding of its effects on cardiac electrophysiology is far from complete.

While single-cell studies have been critical in understanding ion channel physiology, their value in electrophysiological modelling is limited by their lack of intercellular connections. Cardiac tissue slices have been used since the early 1990s to study tissue-level properties (Burnashev et al. 1991). The first study of adult murine heart slices with the MEA was reported in 2006 (Halbach et al. 2006), and adult human slices in 2011 (Camelliti et al. 2011). Several sections can be obtained from the same heart, with relative ease of perfusion and oxygenation. Importantly, tissue structure is maintained within the slice, yet the thinness means propagation can be studied in a pseudo-2D manner.

### Accuracy of Measurements and Chamber Differences

Measured values of ERP, CV and AT were in the expected ranges (see Table 1). For example, Zuberi et al. found murine ventricular ERP to be 38 ms, and Lerner et al. reported a value of 55 ms (Lerner et al. 2000; Zuberi et al. 2010). Using murine hearts perfused with a voltage-sensitive fluorescent dye, Morley et al. found conduction velocities in the range of 30–65 cm s^−1^ (Morley et al. 1999). And Kanai and Salama found LATs of 0–3 ms using guinea pig hearts (Kanai and Salama 1995). Comparison of baseline values across all the experiments revealed highly significant differences between atrial and ventricular tissue for each parameter, with atrial tissue having lower values for all. Such differences were seen in some but not all of the comparisons in individual experiments. It is likely that the larger number of measurements in combined data resulted in greater statistical power to detect differences.

A potential criticism of the study is that ‘horizontal transverse’ or short-axis ventricular slices were used, and myofibre orientation was not delineated in either ventricular nor atrial tissue. The relevance of this is that anisotropy is a well-recognised feature of cardiac conduction, with conduction velocity faster along the long axis compared to transversely across myofibres. Slicing in an alternate plane, to permit stimulation and recording along the long axis of fibres may have resulted in lower variation in ventricular conduction velocities in particular. Also, linear conduction was assumed, though this is almost certainly a simplification of the conduction path. However, we were interested in changes in parameters, rather than absolute values; and this assumption would not have affected measurements of ERP or LAT.

Chamber differences in ERP are fairly well established, at least in larger mammals. Compared with the ventricular action potential, the atrial action potential has a less negative resting potential, an abbreviated plateau phase and slower terminal repolarisation (Fatkin et al. 2007). These differences are predominantly due to increased *I*_to_ and *I*_Kur_ currents, as well as decreased *I*_K1_ current (Fatkin et al. 2007; Koumi et al. 1995), and result in a shorter ERP in atria compared to ventricles. The differences in conduction velocity and activation time may well be accounted for by the differences in connexin expression; in the ventricle being largely Cx43 while in the atria, there is significant expression of Cx40 with Cx43 (Lambiase and Tinker 2015). Cx40 expression has been shown to be inversely associated with atrial conduction velocity (Kanagaratnam et al. 2002).

### Recording Stability and Response

Parameters measured under control conditions without pharmacological or thermal challenge, were stable over 35 min. Ventricular CV did increase significantly, though the magnitude of change was fairly small, (+7 cm/s, *p* < 0.001), equivalent to approximately 16% difference between beginning and end. One possibility for this is a ‘warm-up’ effect, as BDM was fully washed out of the tissue, with restoration of near-normal cellular physiology. In particular, the two main protein complexes involved in impulse propagation, the sodium channel and gap junction complexes, may take longer to recover than other channels.

To assess the responsiveness of the MEA system and its ability to report changes in EP parameters, the sodium channel blocker mexiletine was used. At a concentration of 10^−4^ M, there were significant increases in ERP and LAT, and significant decreases in CV for both atria and ventricles. While myocyte activation may be considered primarily dependent on *I*_Na_, with CV dependent on *I*_Na_ and gap junction conductance, ERP is usually thought to depend on repolarising currents. However, the increases in ERP seen with mexiletine, a pure sodium channel blocker, are probably explained by its preventing re-opening of the sodium channels even after full repolarisation, rather than through effects on the latter. This slowing of the reactivation of sodium channels that normally occurs with repolarisation, prolongs refractoriness independent of action potential duration (APD), which either shortens or remains unchanged with mexiletine (Darbar 2014; Tamargo and Delpon 2014).

### Autonomic Agonists

Atrial ERP shortened with increasing concentration of carbachol, an expected finding likely attributable to activation of *I*_KACh._ In the ventricles, ERP was significantly shorter at 3 × 10^−8^ M, but not at other concentrations. Whether this represents a real effect, or a statistical aberration is difficult to know. It is not concordant with the effects of vagal stimulation in the dog, for example, where lengthening of ventricular ERP occurs (Martins and Zipes 1980). *I*_KACh_ and the corresponding channel subunits (GIRK1 and GIRK4) are thought to be expressed at lower levels in the ventricle (Dobrzynski et al. 2001), so muscarinic agonism would not be expected to prominently alter ventricular ERP in this way. Alternatively, there is evidence to support the idea that Gα_i2_ negatively regulates the L-type calcium channel (Chen et al. 2001; Nagata et al. 2000; Zuberi et al. 2010). A reduction in Ca^2+^ inflow could therefore shorten APD and ERP (Xiao et al. 1999).

Atrial CV did not vary with carbachol, whereas ventricular CV increased. Whether this latter effect reflects a similar process to that occurring in the control experiments, e.g. related to washout of BDM, is unclear. While it is possible that muscarinic signalling exerts effects on *I*_Na_ and thus CV, the absence of effects on LAT for either chamber suggests that this was not the case. Another possibility is that it exerts differential effects on gap junctions, which display some chamber specificity, as described above (Bukauskas 2014; Kléber 2014). More likely, therefore, it would seem to be a type I error.

Isoprenaline had divergent effects on atrial and ventricular ERP. There was a small reduction in atrial ERP, consistent with previous reports (Kim et al. 2012; Olshansky 2005). However, ventricular ERP lengthened slightly, an unexpected finding. For example, Martins et al. showed that in the canine ventricle, ERP shortened in response to sympathetic stimulation, and conversely, sympathetic denervation led to prolongation of ERP (Martins and Zipes 1980). And in humans, the effects are well described, and attributed largely if not wholly to phosphorylation of *I*_Ks_ by protein kinase A (PKA) (Vaseghi and Shivkumar 2008). The resultant increase in K^+^ efflux leads to APD shortening, reflected in the normal reduction in QT interval that occurs with exercise. However, repolarisation in rodents is very different to that in large animals: *I*_Ks_ is not present in the mouse, and the main murine repolarising currents *I*_to_, *I*_Kslow1_, *I*_Kslow2_ and *I*_SS_, are less well studied. In the absence of a βAR-driven increase in K^+^ outflow, βAR agonism may have resulted in an unopposed increase Ca^2+^ inflow producing a prolongation of APD and ERP.

There were no effects of isoprenaline on CV for either chamber, and while there was a small effect on ventricular LAT, no change was seen for atrial LAT. Published data generally support the idea that βAR signalling increases *I*_Na_, via PKA-mediated phosphorylation (Campbell et al. 2014). However, there is not complete agreement (Grant 2009). In terms of functional effects, Lang et al. recently reported that both β1AR and β2AR agonism increased conduction velocity in human cardiac wedge preparations (Lang et al. 2015). And in a review on the subject, Campbell et al. found CV to be increased with βAR stimulation in intact hearts (Campbell et al. 2014). Mechanistically, they suggest that due to the non-linear relationship between d*V*/d*t*_max_ of phase 0 (an *I*_Na_-driven process) and conduction velocity, βAR modulation of gap junction function may also be important, as suggested by previous reports (Burt and Spray 1988). However, most of the work investigating this has been carried out with neonatal cell preparations, without assessment of the effects on conduction. Finally, there may be murine strain differences as in preliminary studies in C57\Black6 mice; we do see an increase (Finlay and Tinker unpublished). This is a topic that needs wider investigation.

### Effects of Temperature on Electrophysiology

Temperature modulation had significant effects of modest to large magnitude on both atrial and ventricular parameters. There was a small, non-significant negative relationship between ERP and temperature for atria, whereas for ventricles, there was a highly significant reduction of large magnitude. CV did not vary with temperature, whereas LAT showed similar patterns to ERP, with a modest negative relationship for atria, and a larger magnitude change for ventricles. Temperature’s relationship with ERP is well studied, and our results are in keeping with previously published work. Spear et al. showed prolongation of activation-recovery intervals with lower temperatures (Spear and Moore 1998), and Coronel et al. found a negative relationship between temperature and monophasic action potential duration in isolated pig hearts (Coronel et al. 2006). Repolarising currents have been shown to exhibit temperature dependence: Kiyosue et al. showed that in guinea pig ventricular myocytes, *I*_K_ was reduced at lower temperatures, and that APD was prolonged (Kiyosue et al. 1993).

The lack of change of CV with temperature was surprising given the reported association. Smeets et al. found an increase in CV with higher temperatures in rabbit atria (Smeets et al. 1986), and Morley et al. found reduction in velocities in mice between 37 and 25 °C (Morley et al. 1999). Two obvious explanations for the results exist. The first is that the temperature range studied was too narrow, and centred around 37 °C. In contrast, the two studies mentioned above had a maximum temperature of 37 °C and a minimum of 25–27 °C. This narrow temperature range was chosen deliberately, as both upper and lower temperatures are quite possible in humans, yet far enough from ‘normal’ to in theory at least, elicit changes. Oddly though, there was not even a non-significant trend, with CV at 34 °C slightly higher than at 37 °C. The second possibility is that murine conduction does not vary so easily with temperature as it does in the rabbit. Finally, the relationship between LAT and temperature is supported by previous experimental work with isolated perfused rabbit hearts (Spear and Moore 1998) and frog ventricular fibres (Hecht 1957). This parameter is dependent on *I*_Na_, and experimental support for the temperature dependence of sodium channel conductance has come from patch-clamp work using rat ventricular myocytes (Milburn et al. 1995).

The importance of temperature in arrhythmogenicity is best exemplified by Brugada syndrome, where fever can trigger ventricular arrhythmias. Dumaine et al. demonstrated a molecular mechanism for the *SCN5A* gene Thr1620Met mutation. They found that current decay kinetics and recovery from inactivation of the channel was altered compared to the wild-type channel (Dumaine et al. 1999). Hypothermia has also been implicated as an arrhythmic trigger, but in the setting of early repolarisation syndrome (Bastiaenen et al. 2010). Although not investigated in this study, arrhythmic triggers are clearly also important. In this regard, Mugelli et al. reported increased after potentials when myocardial temperature is raised from 34 to 37 °C (Mugelli et al. 1986); this was investigated in the context of reperfusion arrhythmias following ischaemia.

### Limitations and Future Directions

We did not check for ischaemia or markers of cell death in the tissue slices, though our technical approach was designed to minimise this, and we closely followed protocols from Camilliti and colleagues who found no evidence for this {ref}. There are a number of interesting possibilities to extend this work. It would be revealing to investigate regional differences in tissue electrophysiology to adapt our methods to compare basal and apical electrophysiology though this was not possible in the current study. It might also be possible to adapt these approaches to study the electrophysiological effects of pathophysiological responses such as ischaemic preconditioning.

## Conclusions

In summary, we have used a multielectrode array system to investigate chamber differences in three important EP parameters, and their responses to autonomic and thermal modulation. The system produced reliable measurements, and was able to report robust changes such as those induced by mexiletine. However, there was some variability between mice and individual preparations. We have demonstrated significant differences in ERP, CV and LAT between atrial and ventricular tissue at baseline. Responses to autonomic agonists may to some degree reflect species differences in signalling and ionic currents, and temperature was shown to have important effects on ERP and LAT.
